# Reporting sterilization as a current contraceptive method among sterilized women: lessons learned from a population with high sterilization rates, Rajasthan, India

**DOI:** 10.1016/j.contraception.2018.10.006

**Published:** 2019-02

**Authors:** Yoonjoung Choi, Anoop Khanna, Linnea Zimmerman, Scott Radloff, Blake Zachary, Danish Ahmad

**Affiliations:** a Johns Hopkins University; b Indian Institute of Health Management Research

**Keywords:** CI, confidence internal, PMA2020, Performance Monitoring and Accountability 2020, Sterilization, Survey, Data quality, Contraceptive prevalence rate

## Abstract

**Objective:**

Measuring current use of contraception relies on self-reported responses from survey respondents. Reporting validity may be affected by women's interpretation of the question and may vary by background characteristics of women. The study aims to understand levels and patterns of underreporting of female sterilization in a population with high sterilization rates.

**Study design:**

Data came from the Performance Monitoring and Accountability 2020 survey conducted in Rajasthan, India, in early 2017. In addition to a conventional question to ascertain current contraceptive use, the survey included a probing question; women who did not report sterilization as a current method were asked if they were ever sterilized. Women were defined as sterilization users based on either question. Among sterilized women, we estimated the percent who reported sterilization as a current method. Multivariable logistic regression analysis was conducted to assess differential reporting across background characteristics.

**Results:**

Among women who were ever sterilized, 78% reported currently using any contraceptive method(s), and 77% reported sterilization as the current method. Women in the lowest household wealth quintile or in general caste were less likely to report sterilization as a current method. Time since sterilization was not associated with correct reporting of sterilization.

**Conclusion:**

This study demonstrates, in a population with high sterilization, that sterilization as a current contraceptive method would be substantially underestimated using conventional survey questions. It highlights the importance of context-specific questionnaire adaptation to measure and monitor contraceptive use and provides implications in measuring current use of contraception in populations with high rates of sterilization.

**Implications:**

The paper examined reporting of sterilization as a current method among sterilized women. Only 77% of sterilized women reported sterilization as a current contraceptive method. In a population with high sterilization, inclusion of a probe question in surveys is recommended to understand reporting quality and accurately measure contraceptive prevalence rates.

## 1 Introduction

The contraceptive prevalence rate is a key indicator to understand and monitor reproductive health in a population. Current use of contraception among women of reproductive age has generally been measured through population-based surveys. The estimates of contraceptive prevalence are based on the responses from a sample of eligible women interviewed by female surveyors. A commonly used question in developing countries is: “Are you or your partner currently doing something or using any method to delay or avoid getting pregnant?” [1], [2].

The validity of such responses has been a concern but assessed relatively rarely. Underreporting of contraceptive use, though not uncommon, largely remains undetected and unreported due to nonavailability of independent data for comparison or verification [3], [4]. The issue of underreporting of contraceptive use gained attention when researchers found discrepancies comparing the responses of men and women [5]. The studies have found underreporting to be a problem specifically in societies where family planning is a sensitive or a stigmatized issue [4], [6], [7]. Evidence suggests not only that underreporting may affect the total prevalence rate but that certain methods may be affected more than others — such as coitally dependent methods [6] and traditional methods [3], [8]. In addition, earlier studies in North India also noted underreporting of contraceptive use, which may be due to women's status within the family and society in conjunction with social norms that are opposed to contraception [9].

Another concern regarding measurement validity arises from use of the word “currently.” Interpretation of the question is left to respondents, without specifying what the time frame of currently is. Women who had not have sex recently (e.g., 2 months ago) may not report using a method currently even if a method was used at the last time she had sex. Meanwhile, women who adopted a permanent or long-acting method in the distant past may not report the method as a current method. The Performance Monitoring and Accountability 2020 (PMA2020) surveys conducted in Rajasthan, India, include a probe question regarding female sterilization and provide a unique opportunity to address the latter aspect of this question.

The study aims to assess the magnitude and pattern of underreporting in current use of sterilization in a population with high sterilization rates. Specific aims are to examine the level of reporting sterilization as a current contraceptive method among women who are sterilized and to assess differential level of correct reporting by background characteristics. Findings from this study will provide implications in measuring current use of contraception through surveys in a population with high rates of sterilization and highlight importance of context-specific adaptation of questionnaires to monitor contraceptive use.

## 2 Methods

### 2.1 Data

PMA2020 is a survey platform that enables frequent and rapid-turnaround monitoring of progress under the Family Planning 2020 initiative. The survey is implemented through resident enumerators equipped with smartphones collecting data every 6 months or annually. Since 2013, PMA2020 surveys have been implemented in 11 countries. It employs a two-stage cluster sampling approach to obtain representative sample of households and women of reproductive age. All women between 15 and 49 years of age in sampled households are eligible for the women's interview. Household and female questionnaires are used to collect data on respondent characteristics, fertility intention, contraception, and other sexual and reproductive health. The questionnaires are adopted from those for the Demographic and Health Surveys to ensure comparability in monitoring trends of key family planning indicators. Detailed information on sampling and survey implementation is available elsewhere [10].

PMA2020 surveys have been implemented in select countries or subnational geographies mostly in sub-Saharan Africa where stakeholders made commitment to accelerate family planning progress under FP2020. In India, the surveys have been implemented only in Rajasthan. Among the countries or subnational geographies where PMA2020 surveys have been conducted, Rajasthan has the highest sterilization rates. The first round PMA2020/Rajasthan survey was conducted between June and September 2016, and quality assurance exercises as well as results indicated underreporting of current contraceptive use especially among women who had been sterilized [11]. It was found that some women did not report sterilization as a current method because they did not consider it a “current” method. It was determined that better ascertainment of sterilization status was critical in the survey population to improve data quality in subsequent surveys. Thus, the second round of PMA2020/Rajasthan — conducted between February and April 2017 — included an additional question to probe sterilization status. Immediately following questions regarding current method use and type of method, the question “Have you ever been sterilized?” was asked to any women who did not report using sterilization as a current method (LCL-301 in Appendix 1) — i.e., women who reported not using any method currently or using a method that is not sterilization. With this additional question, the survey can identify women who may have reported currently not using any method or using a method which is not sterilization but who in fact have been sterilized. Appendix 1 shows a part of the female questionnaire regarding current contraceptive use in the survey. A total of 4994 households and 6041 women between 15 and 49 years completed an interview, with a response rate of 98.3% and 98.3%, respectively. Ethical approval was obtained from the Institutional Review Board at Indian Institute of Health Management Research as well as Johns Hopkins Bloomberg School of Public Health. Datasets are available for the public for research purposes at www.PMA2020.org.

### 2.2 Measures

The main interest of this study is comparing women's sterilization status and reported current contraceptive use. Women were classified as being sterilized if she reported either using sterilization as a current contraceptive method or having been sterilized in the past. A binary variable was created for reporting sterilization as a current contraceptive method.^1^

Additional variables were created to measure background characteristics: 5-year age group; residential area (urban and rural); marital status (currently married vs. not currently married); sexual activity within the last 30 days (yes vs. no); education (none, attended primary school, and attended secondary school or higher); household wealth quintiles; religion (Hindu, Muslim and other); and caste (Schedule caste, Scheduled tribe, other backward class and general). Finally, among those who have been sterilized, the number of years since sterilization was calculated and classified into 5-year incremental groups.

### 2.3 Analysis

The unit of analysis is an individual woman. All analyses were restricted to de facto population (*n*=6015), among women who completed the interview. We first assessed background characteristics by sterilization status (i.e., having been sterilized, regardless of reporting it as a current method) to understand characteristics of those who are sterilized in the study context. Subsequent analyses were then restricted to only sterilized women. We estimated the percent reporting sterilization as a current method by background characteristics and time since sterilization. Differential patterns were assessed using *χ*^2^ test. We conducted bivariate and multivariable logistic regression analyses to estimate the odds of currently reporting sterilization by background characteristics as well as time since sterilization. A p value less than 0.05 was considered statistically significant. All analyses were adjusted for survey sample design, and Stata SE 15 was used.

## 3 Results

### 3.1 Characteristics of sterilized women

Among all women included in our analysis, 30.4% [95% confidence internal (CI): 28.0–32.8] were sterilized — i.e., reported sterilization as a current method or having been sterilized. Compared to nonsterilized women, sterilized women were more likely to be older, more likely to live in rural areas, less likely to be educated and less likely to belong to households in the top wealth quintile (Table 1). Also, the majority of sterilized women were currently married. Among those who were sterilized, women reported being sterilized on average 9.3 years before the interview, and 23% and 46% of the women had been sterilized for 15 or more years and 10 or more years, respectively (Table 1).

**Table 1:** Population characteristics: overall and by sterilization status

Background characteristics	Overall(*n*=6015)	By sterilization status		
	(%)	Not sterilized (*n*=4175) (%)	Ever sterilized^a^ (*n*=1840) (%)	Pearson's *χ*^2^ test p value
Age				
Average (years)	29.0	25.8	36.1	
(95% CI)	(28.7–29.2)	(25.4–26.2)	(35.6–36.6)	
Marital status				
Unmarried	24.9	34.2	3.6	<.01
Married	75.1	65.8	96.4	
Sexual activity in the past 30 days				
No	37.7	46.9	16.5	
Yes	62.3	53.1	83.5	<.01
Residential area				
Rural	64.0	60.8	71.2	<.01
Urban	36.0	39.2	28.8	
Household wealth quintile				
Lowest	16.5	16.6	16.3	<.01
Second lowest	17.5	16.6	19.5	
Middle	19.6	18	23.2	
Second highest	21.5	21.6	21.2	
Highest	24.8	27	19.8	
Highest school attended				
None		26.7	60.3	<.01
Primary	24.4	24.1	25.2	
Secondary or higher	38.7	49.2	14.5	
Caste				
Scheduled caste	22.3	21.4	24.2	.061
Scheduled tribe	17.5	17.2	18.2	
Other backward caste	39.1	38.7	40	
General caste	21.2	22.7	17.6	
Religion				
Hindu	85.3	82.3	92.2	<.01
Muslim	13.3	16.4	6.2	
Other	1.4	1.4	1.6	
Number of years since sterilization				
0–4	N/A	N/A	28.4	N/A
5–9	N/A	N/A	25.7	
10–14	N/A	N/A	22.5	
15–19	N/A	N/A	16.2	
20+	N/A	N/A	7.1	

Analysis restricted de facto population (*n*=6015).

Unweighted number of women. Percent estimates adjusted for sampling weight.

a Women who reported using sterilization currently as a contraceptive method or having been sterilized ever.

### 3.2 Reporting sterilization as a current method: levels and patterns

Among sterilized women (*n*=1840), 77% reported sterilization as a current method, and 78% reported using any modern methods. Reporting of sterilization as a current method was positively associated with household wealth, currently being married and belonging to three caste groups: schedule caste, scheduled tribe or other backward class (Table 2).

**Table 2:** Reporting sterilization as a current contraceptive method by background characteristics, among women who are sterilized (*n*=1840)

	*n*	Not reporting (%)	Reporting (%)	Pearson's *χ*^2^ test p value
Marital status				
Unmarried^a^	64	35.0	65.0	
Married	1776	22.1	77.9	.02
Sexual activity in the past 30 days				
No	306	23.9	76.1	
Yes	1534	22.3	77.7	.66
Residential area				
Rural	1474	21.8	78.2	
Urban	366	24.6	75.4	.63
Household wealth quintile				
Lowest	315	31.0	69.0	
Second lowest	372	24.6	75.4	
Middle	449	22.6	77.4	
Second highest	388	17.2	82.8	
Highest	316	19.4	80.6	.10
Highest school attended				
None	1141	23.0	77.0	
Primary	462	21.2	78.8	
Secondary or higher	237	23.5	76.5	.85
Caste				
Scheduled caste	459	22.5	77.5	
Scheduled tribe	324	20.5	79.5	
Other backward caste	733	19.0	81.0	
General caste	321	33.4	66.6	.08
Religion				
Hindu	1697	22.5	77.5	
Muslim	116	25.2	74.8	
Other	27	18.0	82.0	.86
Number of years since sterilization				
0–4	542	22.3	77.7	
5–9	487	25.1	74.9	
10–14	410	24.5	75.5	
15–19	288	19.6	80.4	
20+	107	15.0	85.0	.24

There was no difference in reporting across the 5-year age groups (results not shown).

Unweighted number of women. Percent estimates adjusted for sampling weight.

a Among sterilized but currently unmarried women (*n*=64), 84% were widowed, 9% were divorced, and 5% were living with a partner.

Adjusted for background characteristics, the odds of correct reporting were higher among those who were currently married than those who were currently not married, of which 84% were widows (odds ratio: 2.3, 95% CI: 1.2–4.4) (Table 3). Also, the adjusted odds of reporting were 46% lower among those in the lowest household wealth quintile compared to those in the three middle quintiles. Women belonging to general caste also had lower odds of reporting than their counterparts. Reporting was not associated with education or urban residence.

**Table 3:** Differential odds of reporting sterilization as a current contraceptive method by background characteristics, among women who are sterilized: bivariate and multivariable logistic regression analyses (*n*=1840)

	Unadjusted odds ratio^a^ (95% CI)	p-value	Adjusted odds ratio^b^ (95% CI)	p value
Marital status				
Unmarried	Reference		Reference	
Married	1.90 (1.10–3.26)	0.02	2.29 (1.19–4.39)	.01
Sexual activity in the past 30 days				
No	reference		reference	
Yes	1.09 (0.73–1.64)	0.66	1.01 (0.61–1.69)	.96
Residential area				
Rural	Reference		Reference	
Urban	0.85 (0.44–1.64)	0.63	0.76 (0.39–1.47)	.41
Highest school attended				
None	Reference		Reference	
Primary	1.05 (0.73–1.53)	0.78	1.07 (0.78–1.47)	.69
Household wealth quintile				
Lowest	0.61 (0.36–1.02)	0.06	0.54 (0.32–0.90)	.02
Middle three quintiles	Reference		Reference	
Highest	1.14 (0.72–1.80)	0.59	1.30 (0.88–1.92)	.19
Caste				
Scheduled caste	1.72 (0.95–3.12)	0.07	1.98 (1.14–3.44)	.02
Scheduled tribe	1.94 (0.87–4.29)	0.10	2.24 (1.05–4.80)	.04
Other backward caste	2.14 (1.24–3.68)	0.01	2.28 (1.32–3.92)	<.01
General caste	Reference		Reference	
Religion				
Hindu	1.07 (0.54–2.14)	0.84	1.08 (0.52–2.26)	.84
Other	Reference		Reference	
Number of years since sterilization				
0–4	Reference		Reference	
5–9	0.86 (0.62–1.19)	0.35	0.94 (0.65–1.36)	.74
10–14	0.88 (0.58–1.33)	0.55	0.97 (0.57–1.64)	.90
15–19	1.18 (0.79–1.77)	0.42	1.24 (0.70–2.20)	.46
20+	1.62 (0.78–3.39)	0.20	1.67 (0.67–4.15)	.27

a Based on regression model including only each characteristic.

b Based on a regression model including all covariates listed in the table and age (5-year groups). Reporting did not vary across the 5-year age groups in either bivariate or multivariable analysis.

Against our hypothesis, reporting was not associated with time since sterilization in either bivariate or multivariable analyses. The null association was confirmed in different multivariable regression models without age and with different categorization of time since sterilization (results not shown).

## 4 Discussion

About 30% of women of reproductive age were sterilized in Rajasthan, India, and only 77% of them reported sterilization as a current contraceptive method. This underreporting of sterilization implies that, among all women, a modern contraceptive prevalence rate would be 36% without the probe question regarding ever sterilization (i.e., solely based on women's report on current methods) but 43% when the ever-sterilized information is utilized (i.e., when the numerator included women who were sterilized but did not report using any methods currently). Among sterilized women, those who belong to households in the lowest wealth quintile or the general caste were less likely to report sterilization as a current method, adjusted for other background characteristics. Women currently unmarried, mostly comprised of widows in our analysis sample, might have not reported using any method since they may not be sexually active and thus do not need to avoid getting pregnant in the study population context. Adjusted for self-reported recent sexual activity, however, the association remained significant. Poor women or women in the general caste might have interpreted certain components of the question differently — such as the time dimension (i.e., “currently”) or need to prevent pregnancy. Meanwhile, women's reporting of sterilization as a current method was not associated with the length of time since sterilization as reported by women.

Measurement of current contraceptive use relies on respondents' reporting quality in population-based surveys. Underreporting of contraceptive use is not uncommon, but it has not been studied extensively, potentially because of the lack of relevant data to investigate the research questions [4]. Nevertheless, various studies have investigated potential underreporting based on disagreement in couple data [5], [12], underreporting by age [13], underreporting where contraceptive use is a sensitive and potentially stigmatized issue [4], [6] and underreporting of traditional methods [3], [8]. Sterilization and, to a lesser extent, other modern methods are sometimes not reported when asked about the method of current use, which may be a result of imprecision in the understanding of the word “current.” It has been suggested that refining the time metric for what entails “current use” would lead to greater accuracy in reporting of contraceptive use [14]. In such cases, interviewers would need to probe while asking questions about sterilization or long-acting reversible methods that women adopted in the past.

Since contraceptive use is considered a sensitive and personal issue, the way the questions are structured, worded and sequenced in the questionnaire may influence the reliability of responses. In National Family Health Surveys in India, departing from the current standard questionnaire for Demographic and Health Surveys [1], the questions related to “ever use” are placed before the questions related to “current use” [15].^2^ In this case, if a woman reports her sterilization when asked about ever use (i.e., “Have you ever used anything or tried in any way to delay or avoid getting pregnant? and “What have you used or done?”), she is not subsequently asked about “current use.” Such sequencing of questions is likely to reduce the inconsistencies and prevents underestimation of sterilization-specific and overall modern contraceptive prevalence rates. The sequencing however does not provide information on the level and pattern of underreporting of contraceptive methods or sterilization. It may also lead to underestimation if interviewers select “no” to ever use to avoid answering additional questions regarding current use later, which can be avoided with training and supervision during the fieldwork, but the questionnaire design should also minimize opportunities for such performance issues.

PMA2020/Rajasthan Round 2 survey introduced a probe question to address this challenge. We found the question critical in capturing 23% of sterilized women, who otherwise would have not been identified. Furthermore, women belonging to the lowest household wealth quintile or general caste were less likely to report sterilization as a current method compared to their counterparts. In a population with high sterilization rate, inclusion of such a probe question should be considered in order to accurately monitor contraceptive use at the population level and understand differential reporting across background characteristics.

## Figures and Tables

**Table:** 

**Section 3: Contraception** Now I would like to talk about family planning — the various ways or methods that a couple can use to delay or avoid a pregnancy. An image will appear on the screen for some methods. If the respondent says that she has not heard of the method or if she hesitates to answer, read the probe aloud and show her the image, if available.		
301a	**Have you ever heard of female sterilization?** PROBE: Women can have an operation to avoid having any more children. [NO IMAGE]	Yes 1 No 0 No response -99
301b	**Have you ever heard of male sterilization?** PROBE: Men can have an operation to avoid having any more children. [NO IMAGE]	Yes 1 No 0 No response -99
301c	**Have you ever heard of the contraceptive implant?** PROBE: Women can have one or several small rods placed in their upper arm by a doctor or nurse, which can prevent pregnancy for one or more years. [IMAGE OF METHOD WILL APPEAR ON SCREEN]	Yes 1 No 0 No response -99
301d	**Have you ever heard of the IUD/PPIUD?** PROBE: Women can have a loop or coil placed inside them by a doctor or a nurse. [IMAGE OF METHOD WILL APPEAR ON SCREEN]	Yes 1 No 0 No response -99
301e	**Have you ever heard of injectables?** PROBE: Women can have an injection by a health provider that stops them from becoming pregnant for one or more months. [IMAGE OF METHOD WILL APPEAR ON SCREEN]	Yes 1 No 0 No response -99
301f	**Have you ever heard of the (birth control) pill?** PROBE: Women can take a pill every day to avoid becoming pregnant. [IMAGE OF METHOD WILL APPEAR ON SCREEN]	Yes 1 No 0 No response -99
301 g	**Have you ever heard of emergency contraception?** PROBE: As an emergency measure after unprotected sexual intercourse women can take special pills at any time within five days to prevent pregnancy. [NO IMAGE]	Yes 1 No 0 No response -99
301 h	**Have you ever heard of male condoms/Nirodh?** PROBE: Men can put a rubber sheath on their penis before sexual intercourse. [IMAGE OF METHOD WILL APPEAR ON SCREEN]	Yes 1 No 0 No response -99
301i	**Have you ever heard of female condoms?** PROBE: Women can put a sheath in their vagina before sexual intercourse. [IMAGE OF METHOD WILL APPEAR ON SCREEN]	Yes 1 No 0 No response -99
301j	**Have you ever heard of the standard days method or Cycle Beads?** PROBE: A Woman can use a string of colored beads to know the days she can get pregnant. On the days she can get pregnant, she and her partner use a condom or do not have sexual intercourse. [IMAGE OF METHOD WILL APPEAR ON SCREEN]	Yes 1 No 0 No response -99
301 k	**Have you ever heard of the Lactational Amenorrhea Method or LAM?** [NO DESCRIPTION; NO IMAGE]	Yes 1 No 0 No response -99
301 l	**Have you ever heard of the rhythm method?** PROBE: Women can avoid pregnancy by not having sexual intercourse on the days of the month they think they can get pregnant. [NO IMAGE]	Yes 1 No 0 No response -99
301 m	**Have you ever heard of the withdrawal method?** PROBE: Men can be careful and pull out before climax. [NO IMAGE]	Yes 1 No 0 No response -99
301n	**Have you ever heard of any other ways or methods that women or men can use to avoid pregnancy?**	Yes 1 No 0 No response -99
302a	[Among women who are not currently pregnant] **Are you or your partner currently doing something or using any method to delay or avoid getting pregnant?**	Yes 1 No 0 No response -99
302b	**[Among women who reported yes in** 302a] **Which method or methods are you using?** **Probe: Anything else?** *Select all methods mentioned. Scroll to the bottom to see all choices.*	Female sterilization 1/0 Male sterilization 1/0 Implant 1/0 IUD/PPIUD 1/0 Injectables 1/0 Pill 1/0 Emergency Contraception 1/0 Male Condom/Nirodh 1/0 Female Condom 1/0 Std. Days/Cycle beads 1/0 LAM 1/0 Rhythm method 1/0 Withdrawal 1/0 Other traditional methods 1/0 No response -99
LCL301⁎	**[Among women who reported no in** 302a or did not report sterilization in 302b] **Have you ever been sterilized?**	Yes 1 No 0 No response -99

⁎ A probe question introduced in the second round of PMA2020/Rajasthan.

## Footnotes

1 A categorical variable was constructed to measure reported current contraceptive method: (1) sterilization, (2) modern methods other than sterilization, (3) traditional methods or (4) none. Exploratory analyses, however, showed that less than 1% of sterilized women reported using modern methods other than sterilization or traditional method. Thus, we decided to examine correct reporting of sterilization with a binary variable.

2 In National Family Health Surveys 1–3 (1992–1993, 1998–1999 and 2005–2006), ever use was asked separately for each method including for sterilization, i.e., “Have you ever had an operation to avoid having any more children?” In National Family Health Surveys 4 (2015–2016), ever use was asked with unprompted responses, i.e., “What have you used or done?”
